# Research culture: science from bench to society

**DOI:** 10.1242/bio.058919

**Published:** 2021-08-11

**Authors:** Lorenzo Canti, Anna Chrzanowska, M. Giulia Doglio, Lia Martina, Tim Van Den Bossche

**Affiliations:** 1 Hematology Research Unit, Groupe Interdisciplinaire de Génoprotéomique Appliquée (GIGA)-I3, University of Liège, 4000 Liège, Belgium; 2 Neuro-Electronic Research Flanders, VIB, 3001 Leuven, Belgium; 3 Biology Department, KU Leuven, 3001 Leuven, Belgium; 4 Interuniversity Microelectronics Centre, 3001 Leuven, Belgium; 5 VIB-UGent Center for Inflammation Research, VIB, 9052 Ghent, Belgium; 6 Department of Internal Medicine and Pediatrics, Ghent University, 9000 Ghent, Belgium; 7 VIB-UGent Center for Medical Biotechnology, VIB, 9052 Ghent, Belgium; 8 Department for Biomolecular Medicine, Faculty of Medicine and Health Sciences, Ghent University, 9000 Ghent, Belgium

**Keywords:** Research culture, Sustainable careers, RRI

## Abstract

Research is a long process in which the collaboration between stakeholders involved in academia, industry and governments is crucial. Ideally, these stakeholders should work together to better align the innovation process with the values, needs and expectations of the research community. Reflecting on how we perform research and how our discoveries can benefit society is therefore of the utmost importance. The complete system of shared values concerning the research process is embedded in the concept of research culture, which has been gaining more attention in recent years. With the hope of increasing awareness of research culture among established scientists and early-career professionals, in this manuscript we discuss what research culture is, what it consists of and how it can positively influence scientific developments.

## Abstract (precis)

**Summary:** With this manuscript, we aim to increase the awareness on research culture, and illustrate that a well developed research culture can positively influence scientific developments.

## The research culture umbrella: an introduction

First, we have to ask ourselves what research culture is, just like Robin Hill in 1999: “Do we mean an organisational culture in which research plays a significant role? Do we mean “the way we do research round here?” Or do we mean a culture of the type found in a petri dish [...] ?” (Hill, 1999). According to the Royal Society of London, research culture encompasses all behaviours, values, expectations, attitudes and norms of research communities. Moreover, it shapes and regulates all aspects of the scientific process: from how research is performed at the bench to how discoveries are communicated to the public (The Royal Society, 2020). Research culture therefore includes a system of shared values and basic assumptions concerning research (Hill, 1999). As a set of common rules, a ‘positive’ research culture emphasises constructive conduct, cooperation and open mindedness, while unhealthy competition and lack of transparency can lead to a ‘negative’ research culture, which will discourage creativity and ultimately hinder scientific progress itself (Anonymous academic, 2018). Luckily, research culture has received increased attention in recent years as a response to the virtual monopoly of the major publishing houses on publications (Larivière et al., 2015), the reproducibility crisis (Dirnagl, 2019) and the increasing use of quantitative metrics to evaluate research outputs (Brown, 2007; Donovan, 2007).

Since the scope of research culture is very broad, we will highlight in this manuscript three different aspects. First, we will discuss the common effort to get ethically acceptable, sustainable and socially desirable research and innovation outcomes by using the Responsible Research and Innovation (RRI) framework. This science policy framework aims to engage academic and industrial entities, the public, political institutions and professionals in science to intimately link and adapt good common practice for aligning with the needs and expectations of society (RRI Practice, 2019) Second, we will discuss gender equality, diversity and inclusivity. Gender equality refers to the given possibility to men and women of all ages to have equal rights in any aspects of their life (EIGE, 2021), something that often goes along with the concepts of inclusion and diversity. These indicate both the organisational effort and practices in which different groups or individuals having different backgrounds are culturally and socially accepted and welcomed, and treated equally. (Global Diversity Practice Ltd, 2020). Third, we will discuss sustainable research careers, which refers to meaningful and beneficial employment to facilitate the personal development of the worker. These aspects will demonstrate how research culture positively impacts the integrity, the openness and transparency of institutions, the diversity and the multidisciplinarity of the research team, and the entire system in which scientists operate (Moore and Jull, 2012).

## RRI: a joint effort to align science with expectations of society

The fast scientific and technological progress starting with the Human Genome Project in 1988 made the need of raising awareness clear about the ethical and social implications of scientific research towards society. In this sense, the RRI framework has to be interpreted as a joint effort to align and reshape science with the expectations of society. Being inspired by the Ethical, Legal and Social Aspects (ELSA) program and other precursors developed all around the world such as the Ethical, Legal and Social Implications (ELSI) program in the USA (1990) and South Korea (2001), and the Genomics-related Ethical, Environmental, Economic, Legal and Social Aspects (GE3LS) program in Canada (2000), the RRI was integrated into Horizon 2020, the most recent European Research and Innovation program promising more breakthroughs, discoveries and world-firsts by taking great ideas from the lab to the market. So, how does this framework find its place within research culture? Founded on the principles of excellence, honesty, moral integrity, transparency and the respect of ethics and professional standards in research (National Research Council et al., 2002), the RRI approach is intended as a progressive framework that embodies all scientific processes, and impacts society at the present and on the long-term (Yu, 2016). Despite few differences, RRI overlaps with the concept of research culture. While research culture is the mere set of values and conducts observed in the context of the scientific and innovation process, RRI is an actual framework showing how science should act towards society (Owen et al., 2013). This framework takes, next to scientists, also other stakeholders into account such as entire research organizations, educational organizations, ethical committees, legislators and the civil society (Stahl, 2013). Each entity benefits from interactions with the other entities. In such a context, communication is of seminal importance: informing stakeholders of the value of the translation process in the innovation chain may make them even more willing to collaborate. From the government's perspective, industry and academics' success in translating and commercializing publicly funded research means the realisation of social and economic value deriving from the discoveries made (Yu, 2016). This in turn justifies further research funding, which researchers and scientific institutions benefit from. Most importantly, the public benefit from the availability of therapies, medicines and technologies derived from funding and human effort (Yu, 2016). In this process of mutual connection, external participants such as international research institutes from abroad can make important contributions as well (Stahl, 2013; Stilgoe et al., 2013). Recent discussions have even focused on how to engage lay citizens in the research process (Heigl et al., 2019) but this is still a matter of debate.

The aim of RRI is thus to understand the role and responsibilities of both the main actors (the scientists) and all participants of the scientific process in Europe. Similar programs have continued or are starting to take place all over the world. Although ELSI is still active from the date of its creation in the USA, this program rapidly expanded from South Korea even to China, Indonesia, Japan, Singapore and Taiwan (Yoshizawa et al., 2014). RRI is a nascent concept in Australia as well (Ashworth et al., 2019), and efforts are being made to engage stakeholders in exporting these concepts in Africa. For example, the non-profit organisation Teaching and Research in Natural Sciences for Development (TReND) in Africa fosters scientific capacity building by acting on all levels (TReND, 2020). First, the organisation supports innovation in local communities by providing tools such as educational training, practical workshops and scientific equipment to allow African researchers to accomplish their own aims. Second, they organise outreach events for the public to inspire locals and to stimulate their interest in science. Finally, to push for stable support for research on the African continent, they also create opportunities for discussions between scientists and local policymakers (politicians and university officers). Such an approach actually promotes opportunities for development and innovation that are integrated and aligned with needs and expectations of society. In this sense, we think the mere re-conceptualisation of issues is not going to help to change perspectives. An overemphasis on individual interests without sufficient attention to the greater social, economic, and structural challenges to translation may undermine rather than protect societal interest (Yu, 2016). On the contrary, letting the stakeholders see the interconnectedness within the chain of translational research and the ultimate objective of public benefit may help them to recognise the importance of their roles and increase participation.

## Gender equality, diversity and inclusion: hand-in-hand for equal opportunities

In spite of the social changes that have happened during the second half of the 20th century, gender inequality persists today and stagnates social progress by hindering collaboration and openness in the work field (Newman, 2014). Addressing such an issue is crucial for ensuring the principle of inclusivity and profit as much as possible by the human capital. Indeed, women represent half of the world's population and therefore also half of its potential. In the science, technology, engineering and math (STEM) workforce, the racial, ethnic and gender gaps are still a major issue. Black and Hispanic workers remain underrepresented, and they are less likely to earn degrees in STEM than other degree fields than Asian and White students (www.pewresearch.org/). Across STEM occupations, women make up a large majority of all workers in health-related jobs, but remain underrepresented in other job clusters, such as the physical sciences, computational sciences and engineering. While gender balance has been reached for degrees in social sciences, biological sciences, mathematics and statistics, women are underrepresented in computer sciences (18.7%), the physical sciences (19.3%) and engineering (20.9%). Additionally, women of color comprise less than 5% of undergraduates in male-dominated STEM fields. Women remain underrepresented at the graduate level, earning 20.1% of doctorates in computer science, 19.3% in the physical sciences, 23.5% in engineering and 28.5% in mathematics and statistics (National Science Foundation et al., 2019). Only 5% of all science and engineering doctorates are awarded to women of color (National Science Foundation et al., 2019). In 2018, the Royal Society of Chemistry showed a worrying steadiness in the number of women retaining leadership positions (Royal Society of Chemistry, 2018a). The report presented evidence that just 9% of chemistry professors in the UK are women, compared to the 44% female undergraduate students, showing how the proportion drops dramatically between undergraduate and senior positions in academia. Actually, the situation in the UK mirrors what happens worldwide, where less than one in three of all research and innovation positions are held by women (UN Woman, 2019). The three key barriers that have to be blamed for such a gender imbalance (Royal Society of Chemistry, 2018b) are (i) relying on uncertain, non-continuous funding, which creates unnecessary pressure, (ii) an inflexible and unsupportive academic culture that can drive talented scientists elsewhere, and (iii) the perception that caring and family responsibilities are unique to women. These barriers not only affect women but also ethnic minorities: evidence from the UK has suggested that poverty and social deprivation in childhood is linked to educational underachievement (Office For National Statistics, 2020). As a proof of concept, white people are 1.5 times more likely to have worked in science than non-white people (The Royal Society, 2014). Setting clear guidelines would be beneficial to supporting the careers of all talented individuals and to ensure an egalitarian approach for everyone. Confronting bias would be a good first step towards building a more meritocratic scientific community. This can be achieved by making people aware of their unconscious biases, i.e. unintended prejudices that influence our perceptions and judgements. To help with the identification of these stereotypes, a team of social psychologists developed the Implicit Association Test which measures an individual's subconscious attitudes, e.g. towards gender and race. Their results showed that 90–95% of people are affected by unconscious biases (Greenwald et al., 1998). This further highlights the importance of pursuing focused training to understand how to recognise and fight such biases. The understanding of unconscious bias could offer an explanation of why society still looks so unequitable despite laws protecting equal opportunities. Another aspect of gender discrimination that is important to keep into consideration is the privilege of men. Privilege can be defined as systematically conferred advantages to a dominant group, which will then have access to resources and institutional power over the outsiders (Bailey, 1998). Namely, it has been seen that men have better chances of getting a job than women, that men are the most represented in the accomplishments highlighting the absence of female role models. Male privilege can be seen even in language, where the masculine words are used to describe a mixed group of people or professions underlining the predominance of the male figure (Dister et al., 2020). The lack of critical interrogation of this privilege allows men to reinforce their dominance and slows down improvements in gender equality (Schacht, 2001). Nevertheless, it is important to recognise that some men are already playing positive roles in fostering equitable gender relations, and such roles must be encouraged and extended at a political level (Chant and Gutmann, 2000). Considering all these aspects and focusing on the specific context of academia, it is critical to find and correct the failure that is impeding the achievement of gender equality. This pitfall could be represented by the system to evaluate researchers' careers that is restricting diversity in academia itself by, for example, penalising women for taking maternity leave or by seeing the maternity leave itself as a lack of productivity. Moreover, researchers should have the possibility to follow leadership courses at work where they can learn how to avoid discrimination and adopt fair and egalitarian leadership styles. The benefits of a diverse and inclusive workforce not only provides social harmony at work for the employees, but also increases productivity and profitability that will help the organisation to succeed (Lean In, 2019).

## Sustainable researcher careers: promoting a positive and supportive research environment

Within such a fast-changing society where the role of an individual is often fulfilled by a successful career path, the topic of sustainable researcher careers is attracting more and more attention. A career is defined as sustainable when it reflects a mutual beneficial interchange between the people and their surrounding environment, and involves the continued employment of individuals in jobs that facilitate their personal development (Vos et al., 2020). Hence, a sustainable career does not refer to success only, but takes into account all of the building blocks for the wellbeing and individual growth of the researcher. Several organisations have been recently involved in understanding whether the scientific career fulfills the requirements of sustainability, highlighting many aspects and habits that still need to change.

The Wellcome Trust conducted a survey involving scientists from different environments, ages and experience who answered that they see their work more as a passion than a job (Wellcome Trust, 2020). This means that researchers voluntarily deal with the deeply rooted habits in the current research culture, including the negative ones. For example, they accept working long hours in conditions of high mental stress and under short-term contracts without security for their future. The current, non-supportive environment ultimately leads to poorer research outcomes (Wellcome Trust, 2020). For most of the surveyed scientists, such compliance is due to the unhealthy competition created by the funding agents' demands for fast and risk-averse results, and the pressure to publish at the expense of individual wellbeing. According to another survey conducted in the Flemish biomedical environment and directed towards various actors (such as policy makers, funders, institutions, editors, but also technicians, students and former researchers), feelings of blame and mistrust are present between groups (Bonn and Pinxten, 2021a,b). In general, these results are reflected in conditions harmful to mental health among scientists, as investigated by the Royal Society. Early-career researchers are considered to be a high-risk group when it comes to work-related stress, with a three times higher risk of developing mental illness than established professionals (Raymer and The Royal Society, 2019). Contrarily, almost all participants of the Wellcome Trust's survey stated the importance of wellbeing for an effective working environment: the best results are achieved when scientists feel a sense of security, the environment is collaborative, inclusive and supportive, and the leadership is transparent and open (Wellcome Trust, 2020). In this regard, institutes and universities need to invest more in services to ensure the wellbeing of researchers. An important role here is played by investigators, supervisors and managers who should be examples of good, ethical research conduct.

Most researchers consider an academic career as the default option and changing career paths is often perceived as failure (Wellcome Trust, 2020). Nevertheless, most researchers' careers will take them outside of academia, according to a joint declaration published by the Marie Curie Alumni Association and the European Council of Doctoral Candidates (Kismihók et al., 2019).

Therefore, academic institutes and governments need to ensure smooth transitions for researchers to non-academic career paths by informing them about these possibilities (Kismihók et al., 2019). Investing in the development of soft skills is a complementary way to increase researchers' employability in and outside academia. Universities are already implementing networking events and transferable skills training in doctoral programs, but also researcher-driven initiatives are very valuable to individual development. Soft skills such as leadership, communication and time management will be considered as essential in at least two thirds of jobs by 2030 (Kismihók et al., 2019). As shown by several reports (Deeming et al., 2017; Gong, 2012; The Royal Society, 2018), the Nuffield Council of Bioethics, 2014) and the progressive increment of academic courses focusing on soft skills, there has been a real increase in awareness of the importance of research culture and its pillars. For example, academic institutes worldwide subscribed to the San Francisco Declaration on Research Assessment (2012) as a sign of recognition of the need to improve the ways in which the outputs of scholarly research are evaluated (DORA, 2012). Current assessment methods do not capture the whole picture of success, which is given by a combination of the researcher, the research outputs, the processes and luck. Moreover, these methods strongly rely on research outputs, discouraging important processes that contribute to the quality and integrity of research (Bonn and Pinxten, 2021a). If publications are relatively easy to track and quantify, how can we evaluate researchers based on their conduct and soft skills? The Royal Society had an idea to create a standardized short format CV that emphasizes wider contributions to the research system, to provide an overview of the individual and the individual's achievements (https://royalsociety.org/blog/2019/10/research-culture/). This format CV is now being piloted by UK Research and Innovation (UKRI), the biggest funder in the UK (UKRI, 2021). Similar innovative ways to evaluate individuals are starting to spread in other countries as well (Tregoning, 2018). Nevertheless, still some years will be needed to bear the fruits of these changes (Saenen, et al., 2021).

In general, there is a clear consensus that a change in today's research culture is essential to ensure the condition of career sustainability in science: healthy competition, openness, mobility (in terms of diversified career paths) and wellbeing must be strongly encouraged and supported. Institutes and universities will have an important role in framing new norms that fulfill the requirements for sustainability, but also researchers themselves have to undergo cultural and attitudinal changes in their own environment and to take responsibility for their own future (Raymer and The Royal Society, 2019).

## Conclusions and future perspectives

Science and technology have tremendous impact on today's society by achieving great discoveries and creating tools from which we can all benefit. At the same time, the current behaviors in terms of competition for funding, low success rates, increasingly long and unstandardised application forms, pressure from the university management to keep the highest level of excellence and so on, put workers' wellbeing and research quality at high risk, leading to suboptimal scientific progression and prosperity of society. Several surveys and reports clearly indicate that a call to action is required: we need to rethink our reward-recognition system and the concept of excellence itself (Moore et al., 2017). These narrow definitions of research excellence suppress researchers' development, creativity and engagement, to the detriment of advances in knowledge and society (Cohen et al., 2019). Therefore, it would be important to promote the reward of applicants and organisations that engage in open and responsible research through multiple tools like offering opportunities for public engagements and showcasing their work on a large scale.

Reimagining research culture needs collective responsibility and requires at the same time continuous evaluation and implementation. Some efforts in this sense have already been made. The Wellcome Trust, for example, launched the Reimagine Research Solutions Summit, a major initiative in September 2019 to bring together experts of the field to explore these topics and think about practical steps to achieve a better research culture. Equally, the global scientific communications company Cactus settled in Asia is driven by its mission to make research available to the community around the world and accelerate research impact (CACTUS, 2021). In this sense, the agency does promote events for enhancing and upgrading research activities of individuals, institutions and funders. Also, it offers a good example of inclusion and collaboration by providing several types of career options, even as a freelancer. The discussion about the different aspects of research culture is open on the web as well: MetisTalk, for example, is a blog that offers a pragmatic way to discuss and put in action the proposed changes in research culture (Downey and Stroobants, 2019).

We can conclude that it is important to leave the narrow reality of the bench, and be more open minded and aware of the impact science and research culture have on the environment we live in, especially for early-career researchers. These changes will allow for bringing different communities from across the research world together, raising the importance of a good research culture and creating ambassadors of change, with people taking the responsibility of going back to their own organisations to stimulate and lead discussions about research culture.
